# 

**Published:** 1881-06

**Authors:** 


### CONTENTS.

### COMMUNICATIONS.

Artificial Crowns ........ 221
Rapid Breathing as a Pain Obtunder in Minor Surgery, Obstetrics, the General Practice of Medicine, and of Dentistry ........ 224

### SELECTIONS.

Leeches........ 246
Another Case of Maternal Impressions, with Singular Malformations ........ 248
Obstetric ........ 249
Causes of Suicide ........ 250
The Treatment of Cancer of the Tongue ........ 250
Bleeding in Tetanus........ 251
Report of Some of the Proceedings of the American Medical Association ........ 352

CORRESPONDENCE........ 254

### EDITORIAL.

Anatomical Charts........ 260
Correction ........ 261
The Eleventh Annual Meeting of the Wisconsin State Dental Society 261
Science Illustrated........ 262
Change of Time ........ 264
The Kentucky State Dental Society........ 264
Special Notice........ 265
Southern Dental Association ........ 265
Alabama Dental Association........ 266
To the American Dental Association........ 266

